# Case report: Recurring and treatment-resistant depression in acquired hepatocerebral degeneration due to a congenital portosystemic shunt

**DOI:** 10.3389/fpsyt.2024.1402695

**Published:** 2024-04-23

**Authors:** Takehiro Tamura, Shunsuke Takagi, Akane Hayakawa, Jun Oyama, Junya Fujino, Hiroki Shiwaku, Hidehiko Takahashi, Genichi Sugihara

**Affiliations:** ^1^ Department of Psychiatry and Behavioral Neurosciences, Graduate School of Medical and Dental Sciences, Tokyo Medical and Dental University, Tokyo, Japan; ^2^ Department of Psychiatry, Tokyo Metropolitan Matsuzawa Hospital, Tokyo, Japan; ^3^ Department of Diagnostic Radiology, Graduate School of Medical and Dental Sciences, Tokyo Medical and Dental University, Tokyo, Japan; ^4^ Center for Brain Integration Research, Tokyo Medical and Dental University, Tokyo, Japan

**Keywords:** acquired hepatocerebral degeneration, shunt embolization, manganese, congenital portosystemic shunt, depression

## Abstract

**Introduction:**

Acquired hepatocerebral degeneration (AHD) is a neurological condition associated with cerebral manganese (Mn) accumulation caused by portosystemic shunts (PSS), usually because of advanced liver disease. AHD is diagnosed by the identification of T1-weighted brain magnetic resonance imaging (MRI) hyperintensities coupled with the presence of PSS and neurological symptoms. Clinical presentations primarily involve motor dysfunction and cognitive impairment. As a result of the frequently concurrent hepatic encephalopathy, the psychiatric symptoms of AHD alone remain unclear. This report is the first documentation of unique psychiatric symptoms of AHD due to a congenital PSS (CPSS) and suggests the efficacy of shunt embolization in achieving sustained remission of psychiatric symptoms in such cases.

**Methods:**

A 57-year-old Japanese woman presented with recurrent severe depression, pain, and somatosensory hallucinations, along with fluctuating motor dysfunction, including parkinsonism, and cognitive impairments. Psychiatric interventions, including antidepressants, antipsychotics or electroconvulsive therapy, had limited efficacy or did not prevent relapse.

**Results:**

T1-weighted MRI showed bilateral hyperintensity in the globus pallidus. No history of Mn exposure or metabolic abnormalities, including copper, was identified. Furthermore, no evidence of liver dysfunction or hyperammonemia was found. Eventually, a gastrorenal shunt was observed on contrast-enhanced abdominal computed tomography. The diagnosis of AHD due to CPSS was made based on the clinical manifestations and abnormal imaging findings. Shunt embolization was performed, which prevented the relapse of psychiatric symptoms and substantially reduced the T1-weighted MRI hyperintensities.

**Conclusions:**

This case highlights the potential involvement of AHD in adult-onset psychiatric symptoms, even in the absence of liver disease. Furthermore, this case underscores the efficacy of shunt embolization in treating the psychiatric symptoms of AHD due to CPSS.

## Introduction

Acquired hepatocerebral degeneration (AHD) is a neurological condition resulting from the accumulation of toxic cerebral metabolites, mainly manganese (Mn), through a portosystemic shunt (PSS) that circumvents hepatic metabolism (1). AHD is diagnosed based on the presence of a PSS, bilateral a hyperintense globus pallidus on T1-weighted brain magnetic resonance imaging (MRI), and neurological symptoms. The diagnosis of typical cases of AHD is not difficult because it occurs concomitantly with advanced liver disease, such as cirrhosis. However, diagnosis becomes challenging when AHD is caused by a PSS without underlying liver disease. One possible cause of such a shunt is a congenital malformation, namely congenital PSSs (CPSS) (2). Prior reports have only focused on hepatic encephalopathy associated with CPSS and have not detailed AHD due to this malformation (3).

The core pathophysiology of AHD has been linked to manganese-related neurotoxicity in the basal ganglia, leading to motor dysfunction, including parkinsonism, and cognitive impairments (4). Mn-related neurotoxicity can cause psychiatric symptoms, classically referred to as “manganese madness,” as observed in occupational manganism resulting from Mn exposure (1). “Manganese madness” is characterized by various psychiatric symptoms, including emotional lability, mood swings, attention deficits, impulsive and compulsive behavior, sleep disturbances, and hallucinations. Mn-related neurotoxicity is mediated through multiple mechanisms, such as inflammation and oxidative stress, leading to the proposed primary pathophysiology: dysfunction of dopaminergic neurons (5). A recent study found that exposure to low Mn doses, even in nonoccupational settings, can cause psychiatric manifestations, particularly depression (6). Despite the considerable potential of Mn-induced neurotoxicity to elicit psychiatric symptoms, few reports have addressed this psychiatric aspect of AHD. This may be partly because psychiatric symptoms of AHD can be conflated with hepatic encephalopathy associated with hyperammonemia (7).

Essential therapeutic strategies for AHD involve mitigating Mn accumulation. Liver transplantation is the most effective treatment for AHD in cases complicated by liver disease (7). Shunt embolization has emerged as a potential strategy for alleviating Mn accumulation by addressing hepatic clearance failure due to PSSs (8), irrespective of the presence or absence of liver disease. To the best of our knowledge, no prior studies have demonstrated the efficacy of shunt embolization in treating unique psychiatric symptoms of AHD.

This is the first report to describe the psychiatric symptoms of a patient with AHD due to CPSS that cannot be explained by the effects of hepatic encephalopathy. It is also the first to demonstrate the efficacy of shunt embolization for various symptoms of AHD, including psychiatric symptoms. The authors conveyed the significance of this study to the patient and her husband through written and verbal communication and secured their written consent.

## Case description

### Diverse clinical manifestations and high-signal findings in the globus pallidus and cerebral peduncle on T1-weighted MRI

**Figure 1:**
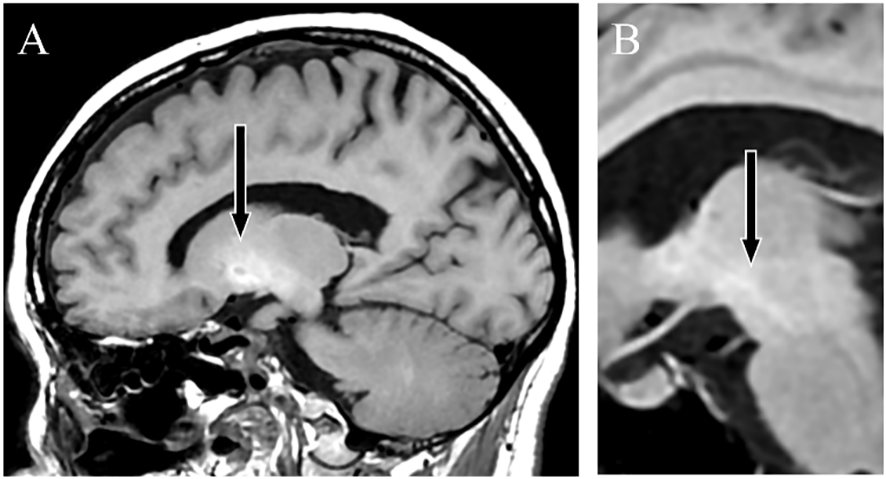
T1-weighted magnetic resonance images. Sagittal sections showing a high-signal intensity in the globus pallidus **(A)** and cerebral peduncle **(B)**, indicating Mn accumulation.

A female patient presented with anxiety and emotional lability, which she had experienced since the age of 40. She had never undergone psychiatric treatment before the age of 50. There was no relevant family history of mental illness. The patient had no psychiatric or medical history, except for appendicitis. At age 50, she reported experiencing pain like “cardboard is stuck in my mouth and it hurts” and somatosensory hallucinations, such as “the floor moving up and down like a wave, causing vertical body shaking.” At age 52, the patient was hospitalized for moderate depression accompanied by severe pain and somatosensory hallucinations. T1-weighted MRI revealed high signal findings in the bilateral globus pallidus and cerebral peduncles (**Figures 1A, B**). However, the brain MRI findings were not further evaluated and were considered incidental findings, because the patient had no history of Mn exposure, heavy alcohol consumption, or chronic liver disease. The patient was diagnosed with delusional disorder (somatic type) and major depressive disorder, discharged after medication with antipsychotics and antidepressants alleviated her symptoms.

At age 53, the patient relapsed into depression and somatosensory hallucinations and began experiencing walking difficulties and forgetfulness. Neurological examination indicated extrapyramidal symptoms such as bradykinesia and postural tremor, in addition to pyramidal tract signs. The patient exhibited mild cognitive impairment, particularly showing limitations in delayed recall and executing tasks based on verbal instructions. Detailed investigations showed no abnormalities in head computed tomography (CT), striatal dopamine transporter-single photon emission CT, or 123I-metaiodobenzylguanidine myocardial scintigraphy. Electroencephalography revealed no discernible frequency abnormalities or epileptiform discharges. No abnormalities were found in the general blood, cerebrospinal fluid, and urine analyses, as well as those of ammonia (24 µmol/L; normal range 9 µg/mL to 47 µg/mL), Mn (2.0 µg/mL; normal range 0.8 µg/mL to 2.5 µg/mL), iron, copper, and pyruvate metabolism-related substances. These findings ruled out Lewy body disease, hepatic encephalopathy, Mn poisoning, Wilson’s disease, pyruvate metabolism disorders, and neurodegeneration with brain iron accumulation. Specifically, Wilson’s disease was comprehensively excluded due to the absence of marked abnormalities in copper metabolism, as indicated by 24-hour urinary copper, serum ceruloplasmin, and blood free copper levels, along with the lack of liver dysfunction and signal heterogeneity on T2-weighted brain MRI.

### Frequent relapse of treatment-resistant psychiatric symptoms

At age 54, the patient relapsed again, undergoing pain, somatosensory hallucinations, and severe depression with catatonic features. Because of medication resistance, electroconvulsive therapy (ECT) was administered for a course of approximately 10 sessions, leading to the remission of psychiatric symptoms. ECT also mitigated motor dysfunction, particularly extrapyramidal symptoms. Despite the success of ECT in mitigating psychiatric symptoms, relapses occurred every year. These relapses recurred despite maintenance therapy with antidepressants, mood stabilizers, and antipsychotics, ultimately compelling further ECT. The psychiatric symptoms exhibited atypical characteristics, with hypomanic episodes and impulsive behaviors manifesting during the relapse at age 55. The patient’s psychiatric symptoms were recurrent and resistant to treatment, and concomitant with neurological and cognitive dysfunction. Such characteristics prompted a reassessment of the diagnosis, that considered the potential involvement of organic factors related to the initial MRI findings.

### Shunt embolization after AHD diagnosis successfully prevented relapse

**Figure 2:**
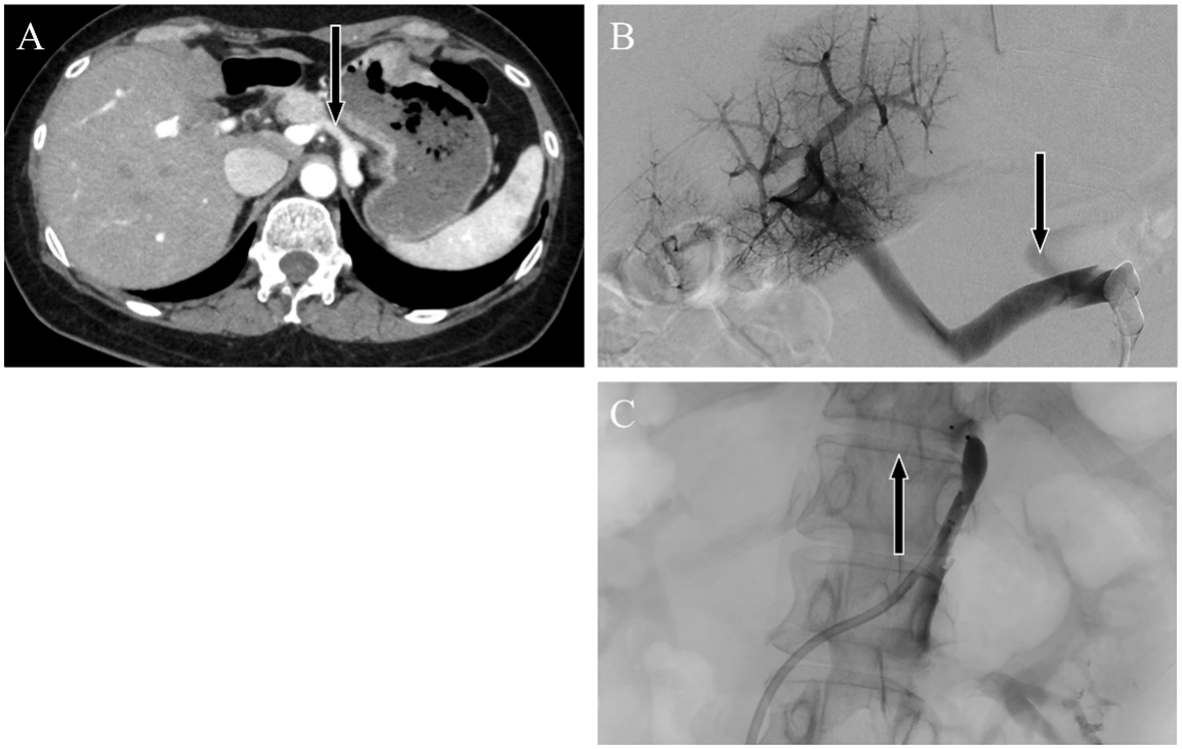
Abdominal dynamic contrast-enhanced computed tomography images. Horizontal sections showing the late arterial phase. Black arrows indicate the **(A)** gastrorenal shunt connecting the left gastric vein to the left renal vein. Angiographic image showing enhancement of the portal vein by contrast injection from a catheter placed in the gastrorenal shunt. Black arrows indicate the portosystemic shunt **(B)** pre-embolization and **(C)** postembolization.

**Figure 3:**
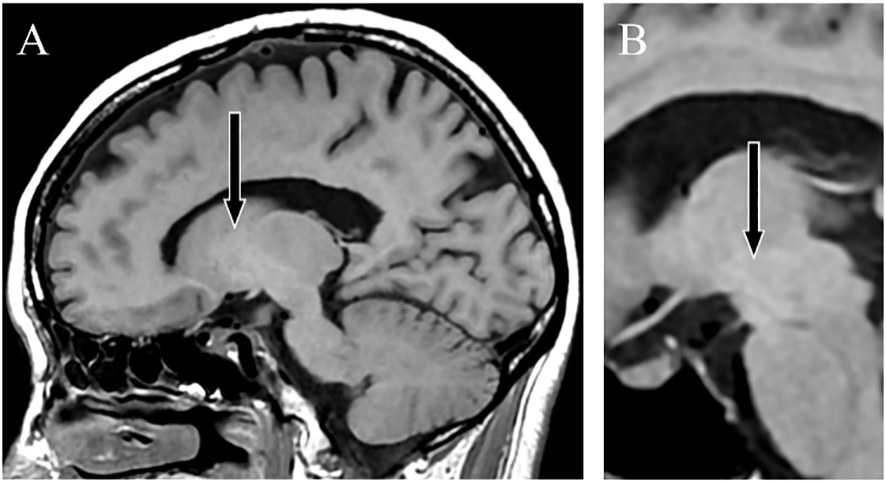
T1-weighted magnetic resonance images. Sagittal sections taken 2 years after shunt embolization showing a lower signal intensity in the globus pallidus **(A)** and cerebral peduncle **(B)**.

**Figure 4:**
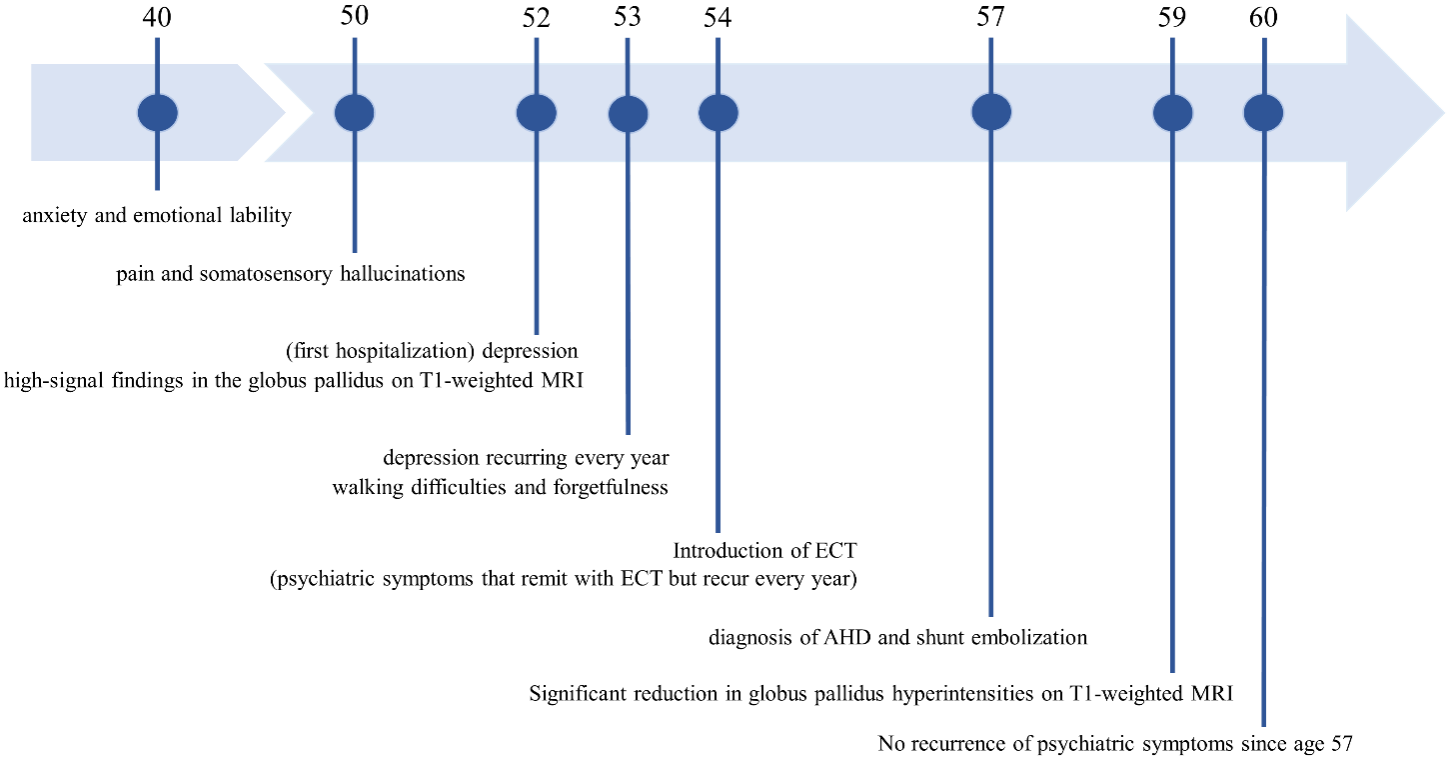
The patient’s disease course and timeline. MRI, Magnetic Resonance Imaging; ECT, Electroconvulsive therapy; AHD, Acquired hepatocerebral degeneration.

Contrast-enhanced abdominal CT revealed a gastrorenal shunt connecting the left gastric vein to the left renal vein shunt that had a diameter of 10 mm (**Figure 2A**). This PSS was diagnosed as a congenital malformation because no underlying conditions were found that could have caused an acquired PSS, such as liver disease or surgical portacaval shunt. Blood transaminase levels and ammonia consistently remained within normal ranges to the extent measurable, which suggested that her symptoms were not caused by hepatic encephalopathy. During the course of the study, Mn concentrations also remained within normal ranges. At age 57, the patient was diagnosed with AHD based on the presence of CPSS, high signal intensity in the bilateral globus pallidus and cerebral peduncles on T1-weighted brain MRI, and clinical symptoms. Digital subtraction angiography revealed the transit of contrast medium from the left renal vein into the portal vein through the gastrorenal shunt, and the absence of portal vein hypoplasia (**Figure 2B**). With the patient’s informed consent, shunt embolization was performed, using 12 mm Amplatzer Vascular Plug I (**Figure 2C**). The shunt embolization procedure proceeded without any notable adverse events. At age 59, follow-up T1-weighted MRI revealed a significant reduction in the hyperintensities in the globus pallidus and cerebral peduncle (**Figures 3A, B**). At age 60, 3 years after shunt embolization, the patient reported no relapses of motor dysfunction or psychiatric symptoms (**Figure 4**). Despite the continued limitations in memory function, there was an overall improvement in her cognitive impairments. She expressed relief at being free of her annual depression and satisfaction with her ability to make routine decisions and solve problems without being overly dependent on her husband.

## Discussion

To the best of our knowledge, this case study is the first to detail the psychiatric symptoms of AHD due to CPSS, without hepatic encephalopathy complications. Furthermore, it documents the efficacy of shunt embolization in sustaining the remission of psychiatric manifestations of AHD due to CPSS. The study also highlights a diagnostic challenge in AHD, particularly in the absence of liver disease, when psychiatric symptoms manifest as the initial clinical presentation.

Psychiatric symptoms associated with PSSs are commonly associated with hepatic encephalopathy but infrequently to AHD. This understanding may stem from the difference between hepatic encephalopathy, which has a high prevalence and distinctive characteristics, such as acute onset altered consciousness and hyperammonemia, and AHD, which has a comparably lower prevalence and manifests with a chronic onset and nonspecific symptoms (7). Moreover, the psychiatric manifestations of AHD may have been previously underestimated because of the co-occurrence of hepatic encephalopathy in the same patient (3). While hepatic encephalopathy and AHD are often discussed as distinct complications of chronic liver disease, some researchers propose that they form part of a single disease continuum, united by a chronic neuroinflammatory process driven by the accumulation of several toxic substances due to hepatic clearance failure (7). This common disease spectrum is also often referred to hepatic encephalopathy, which might have contributed to the less established recognition of psychiatric symptoms associated with AHD. In this study, hepatic encephalopathy is described specifically as a pathological condition characterized by hyperammonemia and acute encephalopathy, primarily involving disturbances in consciousness.

In the present case study, despite the absence of overt disturbances in consciousness, as confirmed by repeated electroencephalography showing normal baseline activity, neurological and psychiatric symptoms persisted. Supported by consistently normal blood ammonia levels, these findings prompted the exclusion of hepatic encephalopathy. A previous case series of AHD with liver disease reported that psychiatric symptoms were observed in 33% of 15 patients with AHD in the absence of hyperammonemia, demonstrating that psychiatric manifestations in AHD are not uncommon (9). Notably, one patient (6.7%) initially manifested psychiatric symptoms solely. These findings confirm that AHD is sometimes difficult to differentiate from psychiatric disorders.

Consistent with observations in the previous case series (9), the present case study reported various psychiatric symptoms, ranging from mild anxiety to catatonia, with major depression emerging as the predominant manifestation. These symptoms are similar to those observed in occupational manganism, which is characterized by the common pathophysiology of Mn neurotoxicity. Despite the absence of elevated blood Mn concentration in the present case, the existing literature indicates that blood Mn concentration is not a reliable indicator of Mn body burden and does not reflect the MRI signal or neurological sequelae of AHD (10). In addition, shunt embolization not only prevented relapse in motor and psychiatric symptoms but also significantly alleviated abnormal brain MRI findings indicative of cerebral Mn accumulation. Therefore, we contend that our case represents a clinical syndrome resulting from Mn-related neurotoxicity. Substantial evidence shows that a pathophysiological mechanism in Mn-induced neurotoxicity is linked to dopamine dysfunction (11), which can induce parkinsonism, psychiatric symptoms, and pain (12, 13).

CPSS, which has an incidence rate of 1 in 30,000, can manifest its initial complications in adulthood, as evidenced by one-third of cases being diagnosed after the age of 12 years, and of some instances reported even beyond the age of 80 years (2). CPSSs are classified according to the presence or absence of an intrahepatic portal vein and the shunt location; type 1 denotes the absence of the intrahepatic portal vein, whereas type 2 indicates its presence (2). The present case was classified as extrahepatic type 2 CPSS. This type of CPSS, where a shunt vessel linking the left gastric vein to the left renal vein converges with the portal vein, is rare. These anatomic features will have restricted the occurrence of complications from CPSS, delaying the diagnosis until middle age. Due to the rare characteristics of this case, the diagnosis of AHD was established 5 years after the identification of distinct MRI features. This case study highlights the importance of considering AHD in adult-onset psychiatric disorders, such as depression, when T1-weighted MRI shows high-signal findings in the globus pallidus, irrespective of the presence of liver disease or elevated blood Mn concentrations.

This case study has several limitations. First, the relationship between the severity of CPSS and the risk of developing complications, particularly the factors causing AHD to manifest after middle age in this case, remains unclear. Second, because of the relatively brief observation period of approximately 3 years after shunt embolization, the assessment of the long-term risk of relapse and impact of shunt embolization on cognitive dysfunction may be premature. Finally, a significant limitation in this case is the inability to rule out the potential involvement of cerebral toxic metabolites, evading hepatic metabolism, other than Mn, in the pathophysiology.

Overall, this case emphasizes the importance of a heightened awareness of AHD due to CPSS in the fields of neurology and psychiatry, especially with hyperintensity in the globus pallidus on T1-weighted MRI. Furthermore, this study has identified the potential of shunt embolization as a highly effective treatment for AHD due to CPSS.

## Data availability statement

The original contributions presented in the study are included in the article/supplementary material. Further inquiries can be directed to the corresponding author.

## Ethics statement

The Ethics Review Committee of the Faculty of Medicine at Tokyo Medical and Dental University stated that ethical review is not required for case reports when individual consent is obtained, and therefore, ethical review was waived for this case study. The studies were conducted in accordance with the local legislation and institutional requirements. The participants provided their written informed consent to participate in this study. Written informed consent was obtained from the participant for the publication of any potentially identifiable images or data included in this article.

## Author contributions

TT: Conceptualization, Data curation, Funding acquisition, Investigation, Project administration, Resources, Supervision, Visualization, Writing – original draft, Writing – review & editing. ST: Investigation, Resources, Supervision, Writing – original draft, Writing – review & editing. AH: Investigation, Resources, Writing – review & editing. JO: Investigation, Methodology, Resources, Supervision, Visualization, Writing – review & editing. JF: Investigation, Resources, Supervision, Writing – review & editing. HS: Investigation, Resources, Supervision, Writing – review & editing. HT: Investigation, Resources, Supervision, Writing – review & editing. GS: Conceptualization, Investigation, Resources, Supervision, Writing – original draft, Writing – review & editing.

## References

[1] Ferrara J Jankovic J. Acquired hepatocerebral degeneration. J Neurol. (2009) 256:320–32. doi: 10.1007/s00415-009-0144-7 19224314

[2] Sokollik C Bandsma RH Gana JC van den Heuvel M Ling SC. Congenital portosystemic shunt: characterization of a multisystem disease. J Pediatr Gastroenterol Nutr. (2013) 56:675–81. doi: 10.1097/MPG.0b013e31828b3750 23412540

[3] Franchi-Abella S Gonzales E Ackermann O Branchereau S Pariente D Guerin F. Congenital portosystemic shunts: diagnosis and treatment. Abdom Radiol (NY). (2018) 43:2023–36. doi: 10.1007/s00261-018-1619-8 29730740

[4] Spahr L Butterworth RF Fontaine S Bui L Therrien G Milette PC. Increased blood manganese in cirrhotic patients: relationship to pallidal magnetic resonance signal hyperintensity and neurological symptoms. Hepatology. (1996) 24:1116–20. doi: 10.1002/(ISSN)1527-3350 8903385

[5] Harischandra DS Ghaisas S Zenitsky G Jin H Kanthasamy A Anantharam V. Manganese-induced neurotoxicity: new insights into the triad of protein misfolding, mitochondrial impairment, and neuroinflammation. Front Neurosci. (2019) 13:654. doi: 10.3389/fnins.2019.00654 31293375 PMC6606738

[6] Racette BA Nelson G Dlamini WW Hershey T Prathibha P Turner JR. Depression and anxiety in a manganese-exposed community. Neurotoxicology. (2021) 85:222–33. doi: 10.1016/j.neuro.2021.05.017 PMC8635218 34087333

[7] Malaquias MJ Pinto CM Ramos C Ferreira S Gandara J Almeida A. Acquired hepatocerebral degeneration and hepatic encephalopathy: one or two entities? Eur J Neurol. (2020) 27:2396–404. doi: 10.1111/ene.14486 32810879

[8] Hisahara S Matsushita T Kitamura M Mezawa S Nonaka M Imai T. Long-term clinical and radiological improvement of chronic acquired hepatocerebral degeneration after obliteration of portosystemic shunt: Report of a case. J Neurol Sci. (2014) 346:303–6. doi: 10.1016/j.jns.2014.07.068 25172193

[9] Klos KJ Ahlskog JE Josephs KA Fealey RD Cowl CT Kumar N. Neurologic spectrum of chronic liver failure and basal ganglia T1 hyperintensity on magnetic resonance imaging: probable manganese neurotoxicity. Arch Neurol. (2005) 62:1385–90. doi: 10.1001/archneur.62.9.1385 16157745

[10] Maffeo E Montuschi A Stura G Giordana MT. Chronic acquired hepatocerebral degeneration, pallidal T1 MRI hyperintensity and manganese in a series of cirrhotic patients. Neurol Sci. (2014) 35:523–30. doi: 10.1007/s10072-013-1458-x 23712371

[11] Criswell SR Warden MN Searles Nielsen S Perlmutter JS Moerlein SM Sheppard L. Selective D2 receptor PET in manganese-exposed workers. Neurology. (2018) 91:e1022–e30. doi: 10.1212/WNL.0000000000006163 PMC6140373 30097475

[12] Tamura T Sugihara G Okita K Mukai Y Matsuda H Shiwaku H. Dopamine dysfunction in depression: application of texture analysis to dopamine transporter single-photon emission computed tomography imaging. Transl Psychiatry. (2022) 12:309. doi: 10.1038/s41398-022-02080-z 35922402 PMC9349249

[13] Jarcho JM Mayer EA Jiang ZK Feier NA London ED. Pain, affective symptoms, and cognitive deficits in patients with cerebral dopamine dysfunction. Pain. (2012) 153:744–54. doi: 10.1016/j.pain.2012.01.002 PMC3816524 22386471

